# Barriers to radiographers' use of radiation safety principles: A qualitative perspective

**DOI:** 10.1002/jmrs.750

**Published:** 2024-01-08

**Authors:** Berit Møller Christensen, Anna Bjällmark, Irine Maghanwi Ndipen, Shilan Shamon Afram, May Bazzi

**Affiliations:** ^1^ Department of Natural Science and Biomedicine, School of Health and Welfare Jönköping University Jönköping Sweden; ^2^ Department of Radiology County Hospital Ryhov Jönköping Sweden; ^3^ Department of Health and Care Sciences University of Gothenburg Gothenburg Sweden

**Keywords:** Diagnostic imaging, patient care, radiographer, research – qualitative, standards

## Abstract

**Introduction:**

To minimise the risks associated with ionising radiation, it is necessary for all staff involved to employ specific techniques to reduce radiation exposure of the patient. These techniques include using compression during examinations of the pelvic region and lumbar spine, using a gonad shield, and asking women if they are pregnant. However, some staff do not use these techniques consistently. Increasing compliance requires determining why staff are non‐compliant. Thus, this study aims to qualitatively investigate why radiographers do not use these techniques.

**Methods:**

This qualitative study is based on a cross‐sectional electronic survey with open‐ended questions. The data were analysed using an inductive qualitative content analysis with quantification of the findings. In total, 111 radiographers from 20 hospitals in Sweden participated.

**Results:**

Three categories appear related to barriers that could obstruct the radiographer from using compression, gonad shields and asking about pregnancy: patient characteristics, interaction between the patient and the radiographer and issues related to the situation and examination.

**Conclusions:**

The barriers to not using radiation protection measures varied depending on the specific measure. However, the barriers were mainly related to the patient experiencing pain, communication difficulties and cultural reasons. In addition, the lack of adequate and user‐friendly equipment was seen as a barrier to applying compression and using gonad shielding.

## Graphical abstract

To minimise the risks associated with ionizing radiation, it is necessary for the radiographer to use radiation dose reduction measures. This qualitative study is based on a cross‐sectional electronic survey with open‐ended questions that were responded by 111 radiographers. The barriers for using dose reduction measures were mainly related to the patient experiencing pain, communication difficulties, and cultural reasons.

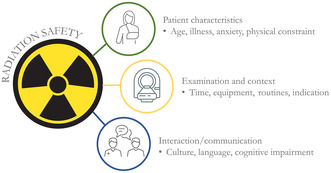

## Background

The use of ionising radiation in medical imaging has raised concerns about its potential harmful effects on patients and staff. To minimise the risks from ionising radiation, all involved staff need to understand radiation exposure and employ techniques for dose reduction and protection. The principles of radiation protection, justification, optimization and dose limitation should guide all medical examinations using ionising radiation. All examinations must adhere to the As Low As Reasonable Achievable (ALARA) principle.^1^ International and national organisations–for example, the World Health Organization (WHO), the International Commission on Radiological Protection (ICRP) and the Swedish Radiation Safety Authority (*Strålsäkerhetsmyndigheten*) (SSM)–work to reduce unnecessary radiation exposure through risk assessment and management. The focus and awareness of radiation protection have increased in recent years,^2^ and more national bodies are now involved in development of standards.^3^

Radiographers are crucial in the performance of radiological examinations. They ensure the well‐being of patients before, during and after diagnostic procedures. In addition, they play an active part in promoting the principles of radiation protection. Compliance with radiation safety guidelines is essential to ensure the safety of the patients and is a main competence of the radiographer profession, which is highlighted in a number of statements internationally and nationally.^4^, ^5^ During the past 10 years, the Swedish Radiation Safety Authority has overseen the implementation of radiation protection routines and guidelines in clinics. Their main focus has been on compliance of radiology departments' routines and guidelines for dose reduction, verifying patient identity and pregnancy status, using gonad shielding for men and using compression during pelvic and lumbar examinations. Several reports from the SSM show a large variety in the degree of compliance between radiology departments as well as large variations over time. In 2018, radiology departments followed their established routines for verifying patient identity in 97% of the examinations, verifying pregnancy status in 83% of the examinations, using gonad shielding in 56% of the examinations and using compression in 58% of the examinations.^6^

Following guidelines is a part of a good patient safety culture. In healthcare, compliance with guidelines and recommendations is influenced by personal willingness, culture, economic and social conditions and levels of knowledge.^7^ Lack of compliance is also related to external factors such as the guidelines, systems and implementation procedures.^8^, ^9^ To the best of our knowledge, no study has identified why radiographers do not comply with patient radiation safety guidelines. Reasons for non‐compliance must be determined to increase compliance. This study aims to qualitatively investigate barriers that keep radiographers from using gonad shields, using compression when examining the pelvic region and lumbar spine and asking women if they are pregnant.

## Methods

### Study design

This study presents the qualitative findings of a cross‐sectional electronic survey, which includes both quantitative and qualitative questions. The electronic questionnaire (Esmaker, Sweden) consists of four parts with a total of 40 questions. The first part concerns questions about the background characteristics of radiographers. The second and third parts present quantitative questions about how radiographers use dose reduction measures as well as factors that influence the use of dose reduction measures. The fourth part asks open‐ended questions where the respondents provide examples of situations that made it difficult for them to use compression, use gonad shields, or ask about pregnancy. This study presents the results from the first and fourth part of the questionnaire. The quantitative findings from the second and third parts are published separately due to word limitations.

The questionnaire was developed collaboratively by the authors and written in Swedish. The questionnaire was tested on three associate professors in radiography with experience in survey construction. Their input was used to ensure that the questionnaire was clear, robust, and aligned with the purpose. The questionnaire underwent minor changes during this process.

In March 2021, the questionnaire and information about the study were distributed to the radiographers via their operation managers. Before the questionnaire was sent to the radiographers, their operation managers phoned or e‐mailed to inform the potential participants about the study and asked them to seek approval from their hospital clinic to participate in the study. The questionnaire was available for 3 weeks and two reminders were sent during this period.

### Study population

The study population was drawn from 466 radiographers from 20 hospitals in Sweden working with conventional radiography or computed tomography (CT). Of the 170 radiographers who agreed to participate in the study, 111 answered at least one of the open‐ended questions and provided background information (Table 1).

**Table 1:** Background characteristics of radiographers (*n* = 111).

Background variable	*n* (%)
**Gender**	
Female	83 (75)
Male	25 (22)
Missing	3 (3)
**Age**	
<35 years	38 (34)
35–49 years	37 (33)
50–65 years	32 (29)
>65 years	1 (1)
Missing	3 (3)
**Working experience as a radiographer**	
<5 years	22 (20)
5–15 years	50 (45)
16–30 years	22 (20)
>30 years	12 (11)
Missing	5 (4)

### Data analysis

The answers to the open‐ended questions were extracted from the questionnaires and were analysed using inductive and manifest qualitative content analysis.^10^ The comments contained in each category were quantified (Table 2). This method combines qualitative and quantitative data.^11^ The combination of qualitative and quantitative data revealed the most frequent barriers to using compression, using gonad shielding and asking about pregnancy status. The researchers involved in the project performed the analysis and discussed the categories until a consensus was reached.

**Table 2:** Categories extracted from the analysis and quantification of the number of comments.

	Compression	Gonad shield	Asking about pregnancy
**Main categories**			
Patient characteristics	Physical constraint (*n* = 13) Age (*n* = 6) Pain (*n* = 45) Anxiety (*n* = 6)	Physical constraint (*n* = 9) Age (*n* = 5)	Serious illness (*n* = 18) Age (*n* = 12)
Interaction between the patient and the radiographer	Language (*n* = 1) Cognitive impairment (*n* = 4)	Language (*n* = 28) Cognitive impairment (*n* = 3)	Language (*n* = 14) Cognitive impairment (*n* = 4) Culture (*n* = 31) Obesity (*n* = 3) Gender change (*n* = 3)
Situation and examination	Indication (*n* = 18) Time (*n* = 8) Equipment (*n* = 5)	Time (*n* = 3) Conceal the information (*n* = 24) Equipment (*n* = 4) Routines (*n* = 7)	–

### Ethical consideration

This study was performed with healthcare professionals and did not involve patients, relatives, or sensitive personal information. Thus, according to the Swedish Ethical Review Act,^12^ no ethical approval was required. This was confirmed by the chair of Jönköping University research ethics committee. Ethical considerations according to the Declaration of Helsinki^13^ were applied. The participants received written information about the study and assurance that participation was voluntary and confidential. Consent to participate was implied by the completion of the questionnaire.

## Results

The qualitative analysis revealed three main categories that describe why the radiographers did not use compression, gonad shielding and pregnancy inquiry: patient characteristics, interaction between the patient and the radiographer and issues related to the situation and examination. The three main categories represent different reasons that hindered the radiographer from using compression, using gonad shielding and asking about pregnancy. For compression, the main barriers were related to patient pain, indication for the examination and physical constraints of the patient. The main reasons for not using gonad shielding were language barriers, a belief that the shieling would conceal important information and physical constraints of the patient. Not asking about pregnancy was mainly related to cultural and language barriers but also to serious illness. Table 2 lists the other reasons for not using compression, not using gonad shielding and not asking about pregnancy.

### Compression

#### Patient characteristics

The radiographers identified patient characteristics—for example, high age, physical constraints and experience of pain and/or anxiety—that affected whether they used compression. Older patients were considered less mobile and sometimes in more pain than younger patients. Movement restriction could also be related to the patient's physique. The radiographers mentioned that procedures with both smaller and larger patients could affect the use of compression equipment. For smaller patients, the radiographers believed that there was nothing to compress; for the larger patients, the radiographers believed the compression equipment was too small to fit them: ‘When the patient is large, it sometimes feels embarrassing for the patient that you need to put on the compression’.

Many radiographers mentioned pain as a factor that could affect whether compression is used as many patients are in pain when they arrive at the radiology department, which makes it difficult to use the compression equipment during the examination: ‘The reason [for not using compression] is that the patients that we examine with conventional abdominal X‐ray are in too much pain to manage compression’. In addition, some radiographers found it difficult to apply the compression equipment on anxious patients because they believed that the anxiety could intensify when the compression is applied: ‘Many patients find it [compression] unpleasant’.

#### Interaction/communication

Several reasons related to communication, such as language and cognitive barriers, could make it difficult for radiographers to explain the use of compression and therefore they avoided using the equipment. Only one of the radiographers mentioned the language barrier, but a few mentioned cognitive barriers such as dementia.

#### Examination and context

The examination and context category includes the examination‐related reasons that keep radiographers from using compression. Specifically, the radiographers mentioned indication, time and equipment as possible barriers. The radiographers working with patients with a possible fracture of the hip or spine avoided using compression: ‘It [using compression] depends on the indication. It can be difficult to use compression when they [the patients] ask about fracture’. Some of the radiographers referred to lack of time as a reason for not using compression. The time aspect was mostly related to the handling of the compression equipment. The compression equipment could be complicated and awkward to use, especially if the radiographer is working alone: ‘If you are the only radiographer in the room, it can be problematic to fasten the compression; compression is awkward to work with’.

### Gonad shield

#### Patient characteristics

The radiographers identified various physical constraints as reasons for not offering the patient a gonad shield. In addition, some patients refused to put on a gonad shield. When fitted with a gonad shield, obese patients could experience discomfort or pain or a patient could find remaining still difficult. Also, radiographers often did not use a gonad shield on unconscious patients or trauma patients on a trauma board: ‘Sometimes, the patient is in pain, and in such cases I do not use a gonad shield. Also, I do not use a gonad shield if the patient is unconscious’. In some cases, the radiographer did not offer a gonad shield to young men because they believed that ‘young men are embarrassed to use a gonad shield’, especially when a relative was in the examination room.

#### Interaction/communication

Barriers related to language were often mentioned as reasons for not offering the patient a gonad shield. In such cases, it was considered that the patient would not understand the information about how to place the shield and the reason for using it and therefore the situation could become awkward. Another obstacle in the communication was cognitive impairment, for example, dementia patients, who could not comply with requests: ‘It is hard to make the patient understand the reason for using the gonad shield and how to place it if we do not speak the same language’. One female radiographer found cultural differences as barriers to applying a gonad shield with a man from another culture. Such situations were often described as difficult, so the radiographer did not offer the shield: ‘Because I am a woman, it feels difficult [offering a gonad shield] to a man from another culture’.

#### Examination and context

Some radiographers considered the use of a gonad shield as time consuming and therefore did not use a gonad shield to save time when the schedule was tight: ‘It [the gonad shield] is time consuming, especially if the patient does not know how to [use it]’. Often, the radiographers did not use a gonad shield on a patient when they considered its use could conceal information needed to interpret the images: ‘If the region around the gonad shield is of interest, then the shield is not used because it can cover what should be seen’. Furthermore, some clinics lacked proper equipment and did not provide shields in various sizes, and specifically shields designed for small children. Also, some radiographers mentioned the lack of routines for using gonad shields, resulting in the radiographers being unsure and not applying a gonad shield when needed: ‘We lack gonad shields designed for small children’ and ‘We do not have sufficient instructions how and when to use gonad shields’.

### Asking about pregnancy

#### Patient characteristics

The radiographers mentioned several barriers related to asking a patient about pregnancy; some of these barriers were related to patient characteristics. Of course, when an unconscious woman came for an acute examination following a trauma, she could not communicate whether she was pregnant and saving her life rather than her pregnancy became the priority: ‘Trauma patients who are unconscious [cannot answer questions]’. Some radiographers also felt uncomfortable asking women who they knew had serious reproductive diseases whether they were pregnant, so they avoided asking ‘women of fertile age who are in treatment for cancer in the reproduction organs’. Also, the woman's age was in some cases considered a reason for not asking about pregnancy. Although the radiographers felt awkward asking older women about pregnancy, they thought it was rather sensitive to ask young women the same question: ‘Very young women often feel embarrassed when asked [about pregnancy] … when the woman is close to the age for being able to get pregnant’.

#### Interaction/communication

Several reasons for not communicating questions regarding pregnancy were related to language barriers such as when the woman and the radiographer did not speak the same language or when the patient was deaf and no interpreter was available. Also, interaction with a patient with a cognitive disability made it difficult to ask about pregnancy due to fears that the patient might not understand the issue: ‘Patients who do not understand Swedish and when an interpreter is not in place’ and ‘when the patient somehow is disabled’.

Several radiographers noted they did not ask a patient about pregnancy if the woman came from another culture even when the patient's husband acted as an interpreter or a teenage woman was accompanied by her parents. In such situations, the radiographer felt insecure asking about pregnancy, avoiding the conversation altogether: ‘Patients where the husband is acting as an interpreter’ and ‘It can be young girls who are accompanied by a parent, and it becomes sensitive to ask about pregnancy’. In addition, some radiographers avoided asking about pregnancy when interacting with a woman suffering from obesity or when the patient had undergone a gender change: ‘If the patient is obese, it can be a sensitive subject’ and ‘Persons who have undergone or are in the process of gender‐change … if they have a uterus’.

## Discussion

Radiographers' perceptions of barriers leading to non‐compliance to radiation protection guidelines varied depending on the type of protection and were related to the patient's characteristics, the examination and the interaction with the patient. A study in the Netherlands about compliance with radiation protection guidelines among radiation technologists found that the overall compliance to guidelines was 59%, although this varied depending on the tasks.^14^ Simons et al.^15^ found that the level of compliance with patient safety tasks was related to the perceived importance of the task. According to Abuzaid et al.,^16^ the experience of the radiographer influenced the use of radiation protection—more experience, higher compliance.

In a literature review of studies from Europe, Asia, Australia and USA, Smiddy et al.^17^ showed that healthcare professional's compliance with guidelines was related to motivational factors and the healthcare professional's perception of the work environment. The motivational factors were influenced by others, and the environmental factors were related to organisational culture, time and knowledge.^17^ Other studies have shown that perceived barriers to non‐compliance were related to the patient's ability and preferences^18^ as well as environmental factors such as equipment and time.^19^

In the present study, patient characteristics (mainly pain) were the main reasons that hindered the use of compression. On the one hand, the radiographer is expected to follow applicable guidelines, such as the radiation protection guidelines; on the other hand, the radiographer is expected to be alert to and alleviate the patient's discomfort and pain.^4^ These requirements place demands on the radiographer and in some situations even lead to dilemmas in the interaction with the patient. The consequences seen in this study indicate that the radiographer may choose to focus on the nursing aspects to ease a patient's pain over the use of radiation protection measures and therefore avoid using compression. Pain measurement instruments have been suggested as a way to evaluate and manage patient pain during radiographic examinations.^20^

Abuzaid et al.^21^ showed that nearly 50% of the radiographers in their study never or sometimes applied gonad shielding. In our study, gonad shielding was not used mainly because of difficulties related to the interaction with the patient, so language was the primary reason for non‐compliance. The radiographer should provide the patient with adequate information related to the radiographic procedure,^4^ but communicative challenges could be an obstacle when informing the patient and applying radiation protection guidelines. Ridelberg et al.^22^ found that the communication between patients and nurses could be both a facilitator and a barrier (e.g. when the patient does not understand the nurse's information) to patient safety culture. Also, a study of nurses in the USA showed communication issues can be a barrier to following guidelines.^9^

A previous study of radiographers in South Africa, a country with 11 official languages, resulted in many recommendations to counter barriers related to cross‐cultural communication between the radiographer and the patient.^23^ Some suggestions mentioned were providing posters and guidebooks containing the most used terms with a translation into other languages. Also, using pictures was mentioned as a tool for facilitating communication.

Barriers to using a gonad shield were also due to the risk that the shield would conceal information. A study in Ireland showed that mispositioning of shielding results in important anatomy being obscured or the gonads not being sufficiently protected.^24^ However, when examining children, the use of gonad shielding was used in only 51% of the cases and 34% of these cases showed inadequate use of the shield.^25^

Some studies have found that non‐compliance with guidelines is often the result of inadequate equipment or lack of equipment.^22^, ^24^ In the present study, the lack of adequate equipment was also a reason for not using gonad shielding or compression. For example, when gonad shields were not available in the right size, the radiographers were non‐compliant. Unfortunately, this barrier is not unique to this study. For example, Doolan et al.^24^ showed that radiology departments often had inadequate supplies of gonad shields. The lack of updated and adequate equipment was also seen as a barrier for nurses to ensure patient safety.^22^ According to Farzanegan et al.,^26^ protective equipment for patients was not used by the radiographers even though it was available in the investigated imaging.

The main barrier for not asking women of fertile age about pregnancy was related to perceived cultural differences between the radiographer and the patient. This situation led to the radiographer not asking about pregnancy. This action could be seen as a way of showing respect for the woman's integrity and cultural expectations. A study in Finland found that nurses believed that good care is based on respect and affirming the patient's culture.^27^

Only a few radiographer's (*n* = 11) mentioned time as a barrier to compliance with radiation protection guidelines. This result may be surprising given that research on other healthcare professions has shown time pressure to be a barrier. Ridelberg et al.^22^ showed that nurses compromised patient safety when they were busy with tasks related to the environment around the patient. Also, studies of practitioners and radiologists have shown time pressure as a barrier to compliance with guidelines in the radiology context.^28^ Similarly, Farzanegan et al.^26^ found that time constraints often led to not using protective equipment for patients.

### Methodological considerations

The subject being investigated is both complex and sparsely studied. Thus, a strength of this study is its design, where qualitative analysis is combined with quantifying comments in each subcategory. This design reveals perceived barriers and the most frequent barriers.

A limitation of this study is that only a few radiation protection measures were studied. In future studies, a more overall approach could be adapted according to the recommendations set by the Swedish Radiation Safety Authority. An interview study could be a way to obtain a more nuanced view of the subject at hand.

Trustworthiness in the study was obtained by considering confirmability, credibility, dependability, and transferability.^29^ Confirmability was obtained by asking open‐ended questions, and the analysis revealed the core aspect of each participant's answers. Credibility was achieved by the researchers contributing their methodological knowledge and experience related to the study design and by discussing the analysis until consensus. Dependability and transferability are considered to be reached to a certain degree. Similar findings would probably be found if carried out in a similar context, so the results could be transferred to a similar context. However, it should be kept in mind that the study provides a cross‐sectional view of the unique circumstances of the data collection.

### Implication for practice

Based on the study results, we believe increasing compliance with radiation safety principles among radiographers requires looking for ways to facilitate using compression, using gonad shields and asking about pregnancy.

As a lack of adequate equipment and instructions for using compression and gonad shielding were mentioned as barriers to compliance, we suggest that the equipment and instructions be updated and readily available. As pain was mentioned as a substantial barrier to not using the equipment, we recommend providing regular shorter clinical education with evidence‐based recommendations regarding pain management of patients. In addition, we recommend using scenario training to help evaluate radiographers' competence and confidence in using the equipment.

As intercultural communication was seen as an obstacle to applying radiation safety principles, we suggest that continuing education is offered, for example, regarding the use of communication tools such as illustrations for various clinical scenarios.

## Conclusion

The radiographer meets a wide range of patients with different abilities and needs and therefore must adjust radiation protection measures to the individual situation. The results of this study show that barriers to not using radiation protection measures vary depending on the specific measure. As the main reason for not using compression was related to patient pain, radiographers should evaluate pain levels using pain assessment instruments and relieve the patient of pain when using compression. According to this study, barriers to the use of gonad shielding and compression are related to the available equipment. Adequate and user‐friendly equipment would probably increase the use of both compression and gonad shielding. This study shows that communication was the main barrier to not using gonad shielding. Hence, education on communication strategies together with the use of different communication tools may improve the communication between the patient and the radiographer. Cultural differences were, according to the present study, barriers to asking about pregnancy, a finding that suggests the need for research that examines how to improve the cultural competency of radiographers.

## Conflict of Interest

The authors declare no conflict of interest.

## Data Availability

The data that support the findings of this study are available on request from the corresponding author. The data are not publicly available due to privacy or ethical restrictions.
